# ABCG2 regulates self-renewal and stem cell marker expression but not tumorigenicity or radiation resistance of glioma cells

**DOI:** 10.1038/srep25956

**Published:** 2016-07-26

**Authors:** Boyoung Wee, Alexander Pietras, Tatsuya Ozawa, Elena Bazzoli, Ondrej Podlaha, Christophe Antczak, Bengt Westermark, Sven Nelander, Lene Uhrbom, Karin Forsberg-Nilsson, Hakim Djaballah, Franziska Michor, Eric C. Holland

**Affiliations:** 1 Cancer Biology and Genetics Program, New York, NY 10021, USA; 2 Brain Tumor Center, New York, NY 10021, USA; 3 Human Biology Division, Solid Tumor and Translational Research, Fred Hutchinson Cancer Research Center, Seattle, WA 98109, USA; 4 Neurosurgery and Alvord Brain Tumor Center, University of Washington, Seattle, WA 98104, USA; 5 Translational Cancer Research, Department of Laboratory Medicine, Lund University, SE-22363 Lund, Sweden; 6 Centro San Giovanni di Dio - Fatebenefratelli, IRCCS, 25123 Bs, Italy; 7 Department of Biostatistics and Computational Biology, Dana-Farber Cancer Institute, Boston, MA 02215, USA; 8 Department of Biostatistics, Harvard School of Public Health, Boston, MA 02215, USA; 9 HTS Core Facility, Memorial Sloan Kettering Cancer Center, New York, NY 10021, USA; 10 Department of Immunology, Genetics and Pathology, Science for Life Laboratory, Rudbeck Laboratory, Uppsala University, 751 85 Uppsala, Sweden

## Abstract

Glioma cells with stem cell traits are thought to be responsible for tumor maintenance and therapeutic failure. Such cells can be enriched based on their inherent drug efflux capability mediated by the ABC transporter ABCG2 using the side population assay, and their characteristics include increased self-renewal, high stem cell marker expression and high tumorigenic capacity *in vivo*. Here, we show that ABCG2 can actively drive expression of stem cell markers and self-renewal in glioma cells. Stem cell markers and self-renewal was enriched in cells with high ABCG2 activity, and could be specifically inhibited by pharmacological and genetic ABCG2 inhibition. Importantly, despite regulating these key characteristics of stem-like tumor cells, ABCG2 activity did not affect radiation resistance or tumorigenicity *in vivo*. ABCG2 effects were Notch-independent and mediated by diverse mechanisms including the transcription factor Mef. Our data demonstrate that characteristics of tumor stem cells are separable, and highlight ABCG2 as a potential driver of glioma stemness.

Stem-like tumor cells are thought to be responsible for tumor maintenance and therapeutic resistance in several cancers^1^,^2^. Studies of such cells rely on experimental isolation from bulk tumor cells, which is most frequently based on expression of stem cell markers such as CD133^3^ and CD44^4^. Stem-like tumor cells display enhanced self-renewal ability and high expression of stem cell markers, both of which have been used as surrogate markers for frequently correlated characteristics that are arguably more important clinically: radiation resistance and tumorigenicity^1^. The precise relationship between these individual characteristics that are collectively referred to as stemness is incompletely understood. Specifically, it is unclear which characteristics are simply correlated markers of stemness and which are drivers of the stem-like phenotype.

One tumor type in which stem-like cells are thought to be of importance is glioblastoma multiforme (GBM), the deadliest of all brain tumors^5^. We recently described one surface marker of stem-like GBM cells – CD44 – that can act as an active driver of stem cell phenotypes including radiation resistance and drug efflux capability in glioma^6^. Thus, stem cell markers may conceivably be attractive therapeutic targets in combination with conventional therapeutics. In the proneural subtype of GBM, as well as in PDGFB-driven murine GBM, cells with properties of cancer stem cells have been functionally enriched based on drug efflux capability using the side population (SP) assay^7^. The SP phenotype - defined as the ability of cells to efflux Hoechst 33342 dye as measured by fluorescence activated cell sorting (FACS) - is dependent on the activity of the ATP-binding cassette transporter ABCG2^8^, which itself may regulate stem cell phenotypes^9^,^10^. SP cells from proneural/PDGFB-driven GBM express higher levels of stem cell markers, display increased self-renewal, increased chemo-resistance, and are more tumorigenic when compared to the sorted main population (MP) cells^7^, but with the exception of chemo-resistance, the contribution of ABCG2 to this phenotype is unclear. Here, we found that ABCG2 function not only correlates with high stem cell marker expression and self-renewal capacity (Bleau *et al*.^7^), but can also actively drive these characteristics of stem-like cells in some GBM cultures. Importantly however, even when enhancing these central features of tumor stemness, ABCG2 activity did not increase radiation resistance or tumorigenicity of such murine or human GBM cells. Gene regulation by ABCG2 was mediated via diverse mechanisms in a cell type-dependent manner, frequently involving the transcription factor *MEF*. In addition to demonstrating a novel and active role for the stem cell marker ABCG2 in driving parts of the glioma stem cell phenotype, our findings emphasize the need to separate the various stem cell characteristics that are usually considered as a collective unit.

## Results

### ABCG2 activity drives expression of stem cell marker genes

**Figure 1:**
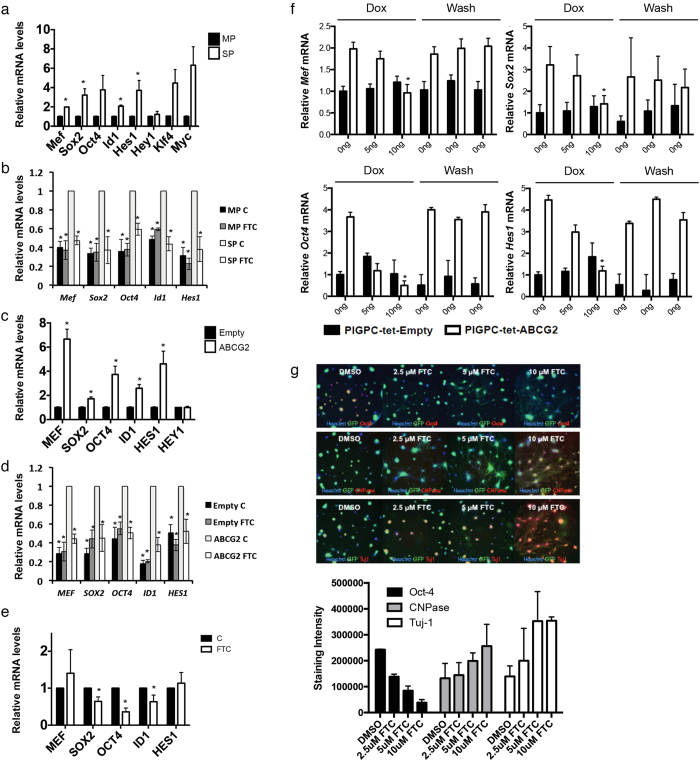
ABCG2 regulates expression of stem cell markers. (**a**) qPCR data of stem cell marker expression in MP and SP sorted PIGPCs. (**b**) qPCR data of stem cell marker expression in MP and SP sorted PIGPCs treated with or without FTC. Statistical comparisons to SP control within each analyzed gene. (**c**) qPCR data of stem cell marker expression in U87-MG-Empty and -ABCG2 cells. (**d**) qPCR data of stem cell marker expression in U87-MG-Empty and -ABCG2 cells treated with or without FTC. Statistical comparisons to ABCG2 control within each analyzed gene. (**e**) qPCR data of stem cell marker expression in primary human U3020-MG glioma cells treated with or without FTC. (**f**) qPCR data of stem cell marker expression in PIGPC-tet-ABCG2 cells treated with or without Dox and washed as indicated. (**g**) Protein expression of Oct4, CNPase and Tuj1 as measured by immunofluorescence and quantified by automated image analysis in PIGPCs. Data represent average values from 3 separate experiments, error bars represent SEM. *p < 0.05.

We sorted side population (SP) and main population (MP) cells from murine PDGF-induced glioma primary cultures (PIGPCs) and measured mRNA expression of a series of established stem cell markers by quantitative real-time PCR (qPCR). SP cells displayed significantly higher levels of 4 out of 8 tested markers, with trends towards higher levels for 3 of the remaining 4 (Fig. 1a), confirming that stem cell marker expression is enriched in SP cells compared to MP cells. To test whether ABCG2 activity was required for this enrichment, we treated sorted SP and MP cells with or without the ABCG2 inhibitor Fumitremorgin C (FTC), and measured expression of the same genes. Intriguingly, for 5 of the 7 genes with higher expression in the SP compared to the MP, FTC treatment reduced expression of these markers in the SP but not in the MP (Fig. 1b), suggesting that ABCG2 may drive at least part of the stem cell phenotype of SP cells. To test the contribution of ABCG2 as a driver of stemness, we generated human U87MG glioma cells overexpressing ABCG2, and isolated SP cells from this line in two steps to ensure a line with high ABCG2 activity (Supplementary Fig. S1). Indeed, expression of 5 of 6 tested markers were significantly higher in U87-ABCG2 cells as compared to U87-Empty cells (Fig. 1c). Similarly to sorted PIGPCs, higher stem cell marker expression in U87-ABCG2 cells was diminished by pharmacological inhibition of ABCG2 activity via FTC, whereas FTC treatment did not affect stem cell marker expression in U87-Empty cells (Fig. 1d). We further found that 3 of 5 stem cell markers were reduced by FTC treatment in the primary human mesenchymal subtype GBM line U3020-MG maintained under stem cell-promoting conditions^11^ (Fig. 1e). Finally, we generated a TetOff inducible system where ABCG2 expression could be rapidly controlled by the addition of Doxycycline (dox) in PIGPCs (Supplementary Fig. S1). Shutting down ABCG2 expression by adding dox decreased expression of all tested genes (Fig. 1f). This repression of expression was reversible, as washing dox away resulted in a rapid re-expression of stem cell markers controlled by ABCG2 (Fig. 1f).

To examine whether ABCG2 affected stem cell markers at the protein level as well, we next treated murine PIGPC SP cells with or without FTC, and stained cells for protein expression of the stem cell marker Oct4 and the two differentiation markers Tuj1 and CNPase. Images were analyzed by automated image analysis quantification. The stainings revealed that Oct4 levels decreased in FTC-treated cells in a dose-dependent manner, whereas expression of Tuj1 and CNPase both were increased by FTC treatment (Fig. 1g). We failed to detect higher levels of the astrocyte marker S100b in FTC-treated cells (Suppl. Fig. S2). High-resolution and magnified images of cells studies are presented in Suppl. Fig. S2. These data are in agreement with the finding that ABCG2 promotes expression of stem cell markers on the gene expression level, and suggest that ABCG2 inhibition may allow stem-like glioma cells to differentiate.

### ABCG2 promotes self-renewal of glioma cells

**Figure 2:**
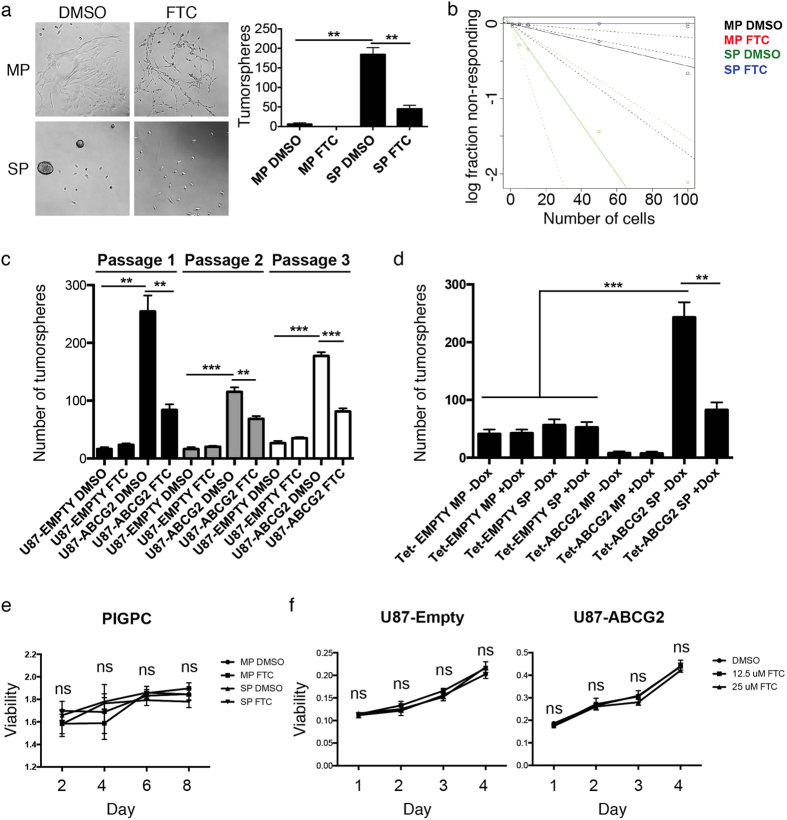
ABCG2 regulates glioma cell self-renewal. (**a**) Sphere forming assay of sorted MP and SP PIGPCs. Data represent number of spheres formed from a minimum of 3 separate experiments, error bars represent SEM. (**b**) Extreme limiting dilution assay of sorted MP and SP PIGPCs treated or not with FTC. 1/(stem cell frequency: 1/24.7 for SP DMSO, 1/176.6 for MP DMSO, and 1/Inf for SP FTC and MP FTC). Dashed lines indicate 95% confidence intervals. (**c**) Sphere forming assay of U87-MG-Empty and -ABCG2 cells treated or not with FTC and passaged 3 times. (**d**) Sphere forming assay of PIGPC-tet-ABCG2 cells treated or not with Dox. Data represent number of spheres formed from a minimum of 3 separate experiments, error bars represent SEM. (**e**) MTT assay to measure viability of sorted MP or SP PIGPCs as indicated, then treated (or not) with 10 μM FTC as indicated. Data represent mean values from 3 independent experiments. Error bars represent SD. (**f**) MTT assay to measure viability of U87-Empty and U87-ABCG2 cells as indicated, treated (or not) with FTC at indicated concentrations. Data represent mean values from 3 independent experiments. Error bars represent SD. **p < 0.01 ***p < 0.001, ns = not significant.

We isolated SP and MP from PIGPCs, seeded them at clonal density under sphere-forming conditions and treated them with or without FTC to inhibit ABCG2 function to measure self-renewal. SP cells formed significantly more spheres than MP cells, and FTC treatment significantly reduced the number of spheres formed by SP cells (Fig. 2a). We subjected cells to the extreme limiting dilution assay (ELDA) and analyzed data using software from http://bioinf.wehi.edu.au/software/elda/12. Cells with high ABCG2 activity displayed a higher frequency of sphere formation compared to those with low or no ABCG2 activity, and the frequency decreased when ABCG2 activity was blocked by FTC in cells with high ABCG2 activity (1/(stem cell frequency): 1/24.7 for SP DMSO, 1/176.6 for MP DMSO, and 1/Inf for SP FTC and MP FTC) (Fig. 2b). Similarly, we seeded human U87-Empty and U87-ABCG2 cells under sphere forming conditions and treated them with or without FTC, then passaged cells serially to measure self-renewal capability while limiting potential effects of differences in adhesion. U87-ABCG2 cells formed significantly more spheres at all passages than U87-Empty control cells, and sphere formation by U87-ABCG2 cells was readily inhibited by FTC (Fig. 2c). By contrast, U87-Empty cells were insensitive to FTC treatment, suggesting that the inhibitory effect of FTC is specific to ABCG2 (Fig. 2c). Finally, in PIGPCs with ABCG2 or empty control expression under Dox control, the addition of Dox significantly reduced sphere formation in Tet-ABCG2 SP but not Tet-ABCG2 MP, Tet-Empty SP or Tet-Empty MP cells (Fig. 2d), again suggesting an ABCG2-specific regulation of tumor cell self-renewal. Importantly, FTC treatment did not affect proliferation of MP or SP PIGPCs (Fig. 2e), or U87-Empty or U87-ABCG2 cells (Fig. 2f), as measured by the MTT assay.

### ABCG2 does not affect response to irradiation or tumor formation *in vivo*

**Figure 3:**
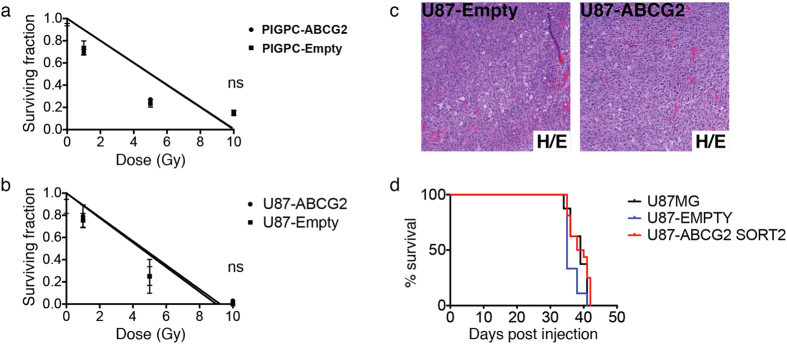
ABCG2 does not affect radiation resistance or tumorigenicity. (**a**,**b**) Colony formation assay following irradiation of indicated cells at indicated doses. Data represent average values of at least 3 independent experiments, error bars represent SEM. (**c**) H/E stainings of U87MG-Empty -ABCG2 intracranial tumors. (**d**) Kaplan-Meier plots for U87MG, U87MG-Empty and U87-ABCG2 tumors. ns = not significant.

One key feature of stem-like tumor cells is resistance to irradiation therapy. We asked whether ABCG2 activity itself promoted radiation resistance of PIGPCs or U87 glioma cells. Cells engineered to overexpress ABCG2 (or controls) were seeded at clonal density, irradiated with 0–10 Gy, then allowed to form colonies over a period of 10 days. Interestingly, ABCG2 expression did not alter radiation response in either PIGPCs or U87 cells (Fig. 3a,b). We and others have previously demonstrated that sorted SP glioma cells more readily form tumors when transplanted into mice than corresponding MP cells^7^,^13^. We next asked whether forced expression of ABCG2, which results in an increased SP phenotype, was sufficient to promote tumor formation. U87 parental cells, U87-Empty and U87-ABCG2 double-sorted cells (Supplementary Fig. S1) were injected intracranially in nude mice, and mice were monitored for tumor formation. The resulting tumors were largely indistinguishable by H/E staining (Fig. 3c) and importantly, there was no significant survival difference between groups (Fig. 3d). Together, these data suggest that while ABCG2 function promotes stem cell marker expression and self-renewal, other key features of stem-like tumor cells may be independently regulated.

### ABCG2 regulation of stemness is Notch-independent and can be mediated by Mef

**Figure 4:**
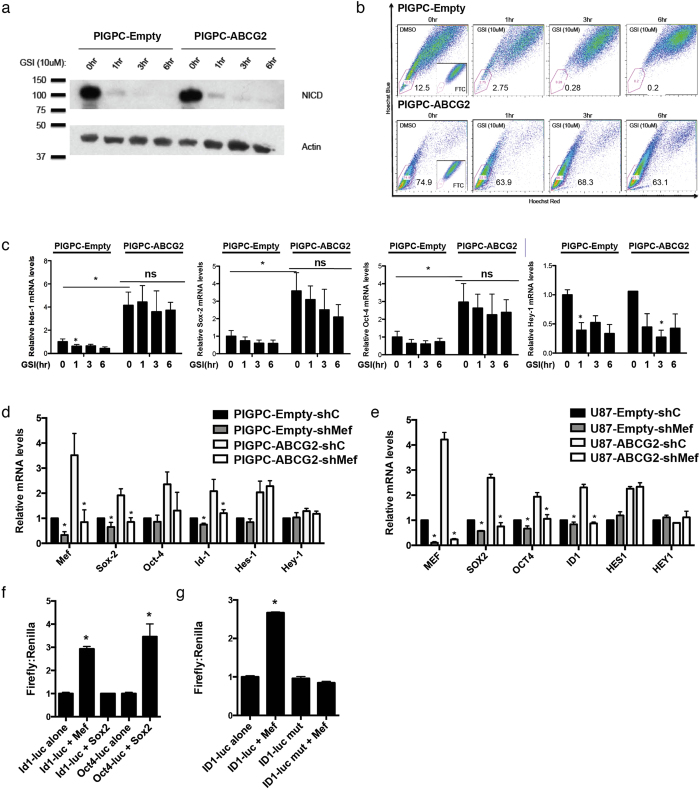
ABCG2 effects are Notch-independent and mediated by Mef. (**a**) Western blot analysis of NICD and Actin in PIGPC-Empty and -ABCG2 cells treated as indicated with γ-secretase inhibitor (GSI). (**b**) SP analysis of PIGPC-Empty and -ABCG2 cells treated as indicated with GSI. (**c**) qPCR analysis of indicated genes in PIGPC-Empty and -ABCG2 cells treated as indicated with GSI. Data represent average values of at least 3 independent experiments, error bars represent SEM. (**d**,**e**) qPCR analysis of indicated genes in PIGPC-Empty and -ABCG2 and U87MG-Empty and -ABCG2 cells treated as indicated with control or *Mef/MEF* shRNAs. Data represent average values of at least 3 independent experiments, error bars represent SEM. Statistical analyzes compared to internal sh controls. (**f**) *Id1-* and *Oct4-*luciferase activity in PIGPCs transfected with the indicated constructs. Data represent average values from 3 separate experiments. Error bars represent SEM. (**g**) *Id1-* and mutated *Id1*-luciferase activity in PIGPCs transfected with the indicated constructs. Data represent average values from 3 separate experiments. Error bars represent SEM. *p < 0.05 ns, not significant.

As Notch is a well-described key player in cancer stem cell regulation, some of the ABCG2-regulated genes (e.g. *Hes1*) are key Notch downstream target genes, and ABCG2/Notch interplay has been previously described^14^,^15^, we speculated that the stemness-promoting effect of ABCG2 could be mediated by Notch. PIGPC-ABCG2 overexpressing cells did not display higher levels of activated Notch intracellular domain (NICD) than PIGPC-Empty controls, and NICD levels were diminished as expected with γ-secretase inhibition in both cell types (Fig. 4a). γ-secretase inhibition had a significant inhibitory effect on the SP phenotype in PIGPC-Empty cells (Fig. 4b), an effect that was largely blocked by ABCG2 overexpression (Fig. 4b). γ-secretase inhibition, as expected, resulted in significantly decreased levels of the Notch downstream target *Hes1* mRNA in PIGPC-Empty cells (Fig. 4c). Intriguingly, γ-secretase inhibition could not significantly diminish *Hes1*, *Sox2*, or *Oct4* levels in ABCG2-overexpressing cells (Fig. 4c), suggesting that ABCG2 regulation of these genes was Notch-independent. Consistent with the above observations, the Notch downstream target gene *Hey1* that was not affected by ABCG2 overexpression was readily inhibited by γ-secretase inhibition in both PIGPC-Empty and PIGPC-ABCG2 cells (Fig. 4c).

We recently described the myeloid Elf-1 like factor (*MEF*) as a novel key transcription factor in promoting glioma stemness and malignancy through direct transcriptional activation of *Sox2*^16^, one of the genes shown here to be activated by ABCG2 in murine and human glioma cultures. We hypothesized that MEF might act downstream to mediate effects of ABCG2 in PIGPCs and U87 cells, and treated PIGPC-Empty and PIGPC-ABCG2 cells with or without shRNA targeting *Mef*. Remarkably, all ABCG2-induced genes except for *Hes1* were insensitive to ABCG2 activation in the absence of *Mef* (Fig. 4d). Similar results were obtained using the U87 human glioma cell line (Fig. 4e), suggesting that indeed, ABCG2 regulation of stem cell marker gene expression (other than *Hes1*) can be mediated by MEF. We previously showed that MEF can directly activate the *Sox2* promoter^16^, and *Sox2* in turn regulates *Oct4* expression. As *Id1* too was activated by ABCG2 in a MEF-dependent manner, we tested activation of an *Id1* promoter-luciferase construct by MEF and Sox2 independently. Intriguingly, the *Id1* promoter was activated by MEF overexpression, while unaffected by Sox2 expression (Fig. 4f), thus suggesting Id1 as a novel MEF transcriptional target gene. To further confirm *Id1* promoter activation by MEF, we mutated a potential MEF binding site in the *Id1* promoter (CGGAA to TTCCG) and transfected cells with MEF. Indeed, this mutated *Id1* promoter was no longer activated by MEF overexpression (Fig. 4g), indicating that *Id1* induction by MEF was direct via MEF binding to the *Id1* promoter. Notably, MEF was not regulated by FTC in U3020-MG cells (Fig. 1e), suggesting that other mechanisms underlying ABCG2-mediated regulation of stemness exist.

## Discussion

Recent studies suggest that a subpopulation of cells with stem-like characteristics may be responsible for glioma repopulation after conventional therapies^17^. Several genes involved in normal stem cell maintenance such as *Mef*, *Sox2*, *Id1*, and *Notch* have been shown to increase malignancy (with or without affecting tumorigenicity) of gliomas^16^,^18^,^19^,^20^. In this study, the role of ABCG2 function in stem cell marker maintenance and sphere formation was examined. Cells with high ABCG2 activity show increased levels of transcripts that are involved in stemness such as *Mef*, *Sox2*, *Oct4*, *Id1*, and *Hes1*, and the activity of ABCG2 is required for maintaining these stem markers. Interestingly, Mef was required for *Sox2* and *Id1* regulation in primary murine and human U87 glioma cells, both of which are important in maintaining the balance between differentiation and self-renewal. The ABCG2-dependent activation of *Hes1* was neither Mef-dependent nor Notch cleavage-dependent. Conventionally, Notch signaling is believed to be upstream of ABCG2 function and expression^21^,^22^. The fact that *Hey1*, another readout of Notch signaling was affected by neither ABCG2 function nor Mef suggests that ABCG2 activity does not regulate these transcription factors through Notch.

Stem cell markers like *SOX2* and *ID1* were regulated by ABCG2 in a primary human GBM line despite *MEF* not being regulated in these cells. These findings together suggest that ABCG2 may regulate stemness in a context-dependent manner, sometimes in a MEF-dependent pathway, and sometimes in a MEF-independent manner. It is further likely in light of the present investigation and previous studies that not all GBM tumors display the side population phenotype and thus ABCG2 function^23^. Whether such tumors are less stem-like than those that do remain an open question, but we have noted sustained self-renewal and stem cell marker expression even in cells derived from samples lacking the side population phenotype^11^.

A great deal has been made of the ability for tumor cells to form spheres in culture, however it is not clear what this phenomenon really means with respect to the behavior of tumors *in vivo*^24^. Regardless of its importance in tumor biology, the sphere-forming ability is downstream of Sox2^16^. The fact that 2 of the 4 iPS factors that covert terminally differentiated cells into stem cells are elevated by ABCG2 activity could suggest that it may have the capacity of lowering the threshold for acquiring full stem cell characteristics. However, although the other 2 iPS factors are higher in cells with high ABCG2 activity, the are not regulated by ABCG2, implying ABCG2 is unlikely to actually achieve full stem cell characteristics on its own. This is certainly the case in our tumor models, since the clinically critical stem cell characteristics of *in vivo* tumor formation and radiation resistance were not affected by ABCG2 function. Notably, we previously published increased chemo-resistance of ABCG2-expressing cells to some chemo-therapeutic agents. These effects are likely directly related to the function of ABCG2 as a drug efflux pump. Together, our data imply that some of the characteristics collectively associated with cancer stem cells are in part separable. It also suggests that the elevated levels of expression of these specific markers and sphere formation are not direct drivers of aggressive tumor behavior in glioma, but rather correlated biomarkers for that behavior.

Many cell surface markers for stem cells have been identified for their use in enriching living cell populations with stem cell characteristics. Most of these markers are likely to correlate with stem cell behavior rather than being drivers of it. However, ABCG2, like CD44^6^, is correlated with stem cell behavior in tumor cells because it can actively drive some of the characteristics that define these cells. One might guess that a driver of stem cell characteristics would be a good therapeutic target. However, this is unclear given that ABCG2 appears not to regulate the components of stem cell character that lead to therapeutic resistance and recurrence. It is possible that effects measured on self-renewal and stem cell marker expression by ABCG2 are too modest to affect these other stem cell characteristics. This possibility does not, however, alter the conclusion that self-renewal and stem cell marker expression outcomes may need to be separated from tumorigenicity and radio-resistance.

Finally, further work is needed to determine whether the pumping activity of ABCG2 itself or interactions between ABCG2 and other membrane proteins is important in the regulation of stem cell markers and sphere formation by ABCG2.

## Methods

### Cell culture

RCAS/tv-a system has been described in previous studies^25^,^26^. PDGF-induced gliomas were dissected and enzymatically digested for 10 minutes in EBSS solution containing 12% papain (Worthington) and 10 μg/ml DNase at 37 °C. The digestion was stopped using 1 mg/ml ovomucoid (Worthington) dissolved in basal neural medium containing 10 μg/ml DNase. Cells were washed using basal neural medium three times and plated in DMEM containing 10% FBS. Only the cultures between passage numbers p0 and p2 were used in the study. U87-MG cells were obtained from ATCC. U3020-MG is a primary human GBM line from HGCC (www.hgcc.se), as previously described^11^. To generate ABCG2-expressing cultures, Platinum E packaging cells were transfected with retroviral constructs, pbabe-EMPTY-puro and pbabe-ABCG2-puro, that were previously described^7^ using Fugene (Roche). Viral supernatants were then harvested 24 hrs after transfection and filtered using 0.45 μm filter, and cells were infected for two cycles for two days in the presence of 8 μg/ml of polybrene. Cells were recovered for 24 hrs or until they reached 70–90% confluency with fresh medium, and then selected with 1 or 2 μg/ml puromycin depending on cell lines. Cell viability was measured by the MTT assay (Roche) as per the manufacturer’s recommendations.

### Hoechst 33342 staining and flow cytometry

Cells to be analyzed were suspended at 2*10^6 ^cells/ml in Neural Stem Cell (NSC) basal medium and incubated at 37 °C for 30 minutes with or without Fumitremorgin C (FTC), a specific ABCG2 inhibitor (Sigma) or gamma secretase inhibitor (GSI)-MK-003, a kind donation from Merck, USA. Cells were then incubated for 90 minutes at 37 °C with 5 mg/ml Hoechst 33342. After the staining, cells were incubated on ice for 10 minutes and washed with ice-cold PBS. Hoechst dye was excited at 407 nm by trigon violet laser, and dual wavelengths were read using 450/40 (Hoechst 33342-Blue) and 695/40 (Hoechst 33342-Red) filters. Dead cells were excluded by gating on forward and side scatter and by eliminating propidium iodide (PI)-positive population. The results were analyzed using FlowJo (Ashland, OR).

### Sphere formation assay

Mouse primary cultures as well as human U87MG cells cultured in DMEM + 10% FBS were washed thoroughly and plated at clonal density (5 × 10^3 ^cells/per well in 6-well plates for regular sphere formation and 100, 50, 10, 5, 1 cells/per well in regular 96-well plates for Limiting Dilution Assay) in medium containing neural stem cell (NSC) basal medium, NSC proliferation supplements, 10 ng/ml EGF, 20 ng/ml basic-FGF, and 1 mg/ml Heparin (Stem Cell Technologies) either with DMSO or 10 μM FTC. Number of spheres was counted after 1 week.

### High-throughput Assay and Automated Imaging

5 μL DMSO control or 12 doubling dilutions of FTC were preplated in 384-well format at 10× concentration to reach up to 10 μM FTC in 1% DMSO (v/v) final concentration. Following cell sorting, 4,000 cells were seeded per well in 45 μL media and cells were imaged live on the GFP channel every 24 hours over 19 days. Compound or DMSO control was replenished on day 8, and on day 12 and 18 in combination with fresh media. At day 19, cells were fixed with 4% PFA (v/v) in PBS for 20 minutes, washed with PBS, and nuclei were stained and cells were permeabilized with a solution of 10 μM Hoechst and 0.05% Triton X-100 (v/v) in PBS for 10 minutes, followed by one wash in PBS. Cells were blocked for 1.5 hours at room temperature with a solution of 10% FBS in PBS (v/v). After aspiration, a 1:40 dilution of the primary antibodies - Oct-4 (Abcam), CNPase (Millpore), and Tuj-1 (Millpore) - in 1% FBS in PBS (v/v) was incubated with cells for one hour at room temperature. Following two washes in PBS, a Alexa Fluor 647-conjugated secondary antibodies (Molecular Probes) in 1% FBS in PBS (v/v) was incubated with cells for one hour at room temperature. After two washes in PBS, the plate was sealed and imaged. For live imaging and whole well quantification of Oct-4, CNPase, and Tuj-1 staining, whole well images were acquired using the IN Cell Analyzer 2000 (INCA2000) automated epifluorescence microscope (GE Healthcare) at 4× magnification as previously described^27^. Automated image analysis was conducted with the IN Cell Developer 1.9 software (GE Healthcare) using custom-developed analysis protocols. Images in the DAPI channel were acquired using 350/50 nm excitation and 455/50 nm emission filters with a 0.1 second exposure time. Images in the FITC channel were acquired using 490/20 nm excitation and 525/36 nm emission filters with a 0.5 second exposure time. Images in the Cy5 channel were acquired using 645/30 nm excitation and 705/72 nm emission filters with a 1.5 second exposure time. High resolution confocal microscopy image were captured using an IN Cell Analyzer 3000 (INCA3000) automated confocal microscope (GE Healthcare) as previously described^28^. Images were acquired at 364 nm excitation and 450/65 nm emission for Hoechst, at 488 nm excitation and 535/45 nm emission for GFP, and at 633 nm excitation and 695/55 nm emission for immunostaining of Oct-4, CNPase, and Tuj-1 with Alexa Fluor 647-conjugated secondary antibodies (Molecular Probes). Images were captured with an exposure time of 1.5 ms, collecting 9 images per well at 40× magnification. Data was acquired and processed using the Raven 1.0 software (GE Healthcare).

### Luciferase assay

Cells were co-transfected with pCMV-Renilla along with following plasmids: 1.5BV Id1-luciferase (kind gift from Dr. Benezra) and Oct-4 luciferase (described in^16^). MEF binding site were mutated using QuikChange II XL Site-Directed Mutagenesis Kit (Agilent technologies). Following primers were synthesized using a software from https://www.genomics.agilent.com/ for site-directed mutagenesis:

mId1mt-F: 5′-agttcatttctctagaaatttgagtcaggcattccgttcagagtaaaagagagcctgctttgaaatc-3′ mId1mt-R: 5′-gatttcaaagcaggctctcttttactctgaacggaatgcctgactcaaatttctagagaaatgaact-3′.

The luminescence of the lysates was measured using Dual-Luciferase Reporter Assay System (Promega) and read by Veritas microplate luminometer (Turner Biosystems). The Firefly luminescence was normalized to Renilla luminescence.

### Antibodies

Primary antibodies - ABCG2 (1:200, Abcam), NICD (1:500, Cell Signaling), and Actin (1:8000, Millpore) - were dissolved in blocking reagent and incubated overnight at 4 °C. Horseradish peroxidase-conjugated secondary antibodies - anti-rat HRP (GE Healthcare), anti-rabbit HRP (GE Healthcare), and anti-mouse HRP (Roche) - were used and then visualized using ECL chemiluminescence (GE Healthcare).

### Colony formation assay

Mouse primary cultures as well as human U87MG cells of differing ABCG2 activities as indicated, cultured in DMEM + 10% FBS, were seeded at clonal density (5 × 10^2 ^cells/per well in 6-well plates). Cells were seeded at Day1 and radiated 0, 1, 5, 10 Gy at Day 2. 10 days after radiation, colonies were stained with crystal violet and counted.

### Statistical Analysis

Two-tailed t tests were used to analyze data from qRT-PCR and luciferase assays. Data represent the mean of three independent experiments unless otherwise noted. p values of <0.05 were considered statistically significant.

### *In vivo* tumorigenicity assay

Newborn NOD-SCID pups were intracranially injected with 1 × 10^5^ cells of differing ABCG2 activities as indicated (Supplementary Fig. S1), using a Hamilton syringe. Mice were carefully monitored and euthanized when symptoms such as lethargy and cachexia developed. All procedures were approved by and carried out in accordance with guidelines approved by the Institutional Animal Care and Use Committee at Memorial Sloan-Kettering Cancer Center (protocol# 00-11-189).

### Real-time qPCR analysis

RNAs were purified using miRNeasy extraction kit (Qiagen) and cDNAs were synthesized using Superscript III First-Strand Synthesis system for reverse-transcriptase PCR (Invitrogen). Real-time PCR was performed on the 7900HT Fast Real Time PCR System (Applied Biosystems) using primers listed in Supplementary Table S1.

## Additional Information

**How to cite this article**: Wee, B. *et al*. ABCG2 regulates self-renewal and stem cell marker expression but not tumorigenicity or radiation resistance of glioma cells. *Sci. Rep.* **6**, 25956; doi: 10.1038/srep25956 (2016).

## Supplementary Material

Supplementary Information
