# Supplementary material for: ABCG2 regulates self-renewal and stem cell marker expression but not tumorigenicity or radiation resistance of glioma cells
Source: Sci Rep. 2016 Jul 26;6:25956. doi: 10.1038/srep25956 (PMC4960591; doi:10.1038/srep25956)
Supplement: Supplementary Information [file srep25956-s1.pdf]

Supplementary information

ABCG2 regulates self-renewal and stem cell marker expression but not tumorigenicity or radiation resistance of glioma cells

Boyoung Wee[1,2,11], Alexander Pietras[3,4,5,11], Tatsuya Ozawa[3,4], Elena Bazzoli[1,2], Ondrej Podlaha[6,7], Christophe Antczak[8,9], Bengt Westermark[10], Sven Nelander[10], Lene Uhrbom[10], Karin Forsberg-Nilsson[10], Hakim Djaballah[8], Franziska Michor[6,7], and Eric C. Holland[3,4]

[1]Cancer Biology and Genetics Program, [2]Brain Tumor Center, [8]HTS Core Facility, Memorial Sloan Kettering Cancer Center, New York, NY 10021, USA; [3]Human Biology Division, Solid Tumor and Translational Research, Fred Hutchinson Cancer Research Center, Seattle, WA 98109, USA; [4]Neurosurgery and Alvord Brain Tumor Center, University of Washington, Seattle, WA 98104, USA; [5]Translational Cancer Research, Department of Laboratory Medicine, Lund University, SE-22363 Lund, Sweden; [6]Department of Biostatistics and Computational Biology, Dana-Farber Cancer Institute, Boston, MA 02215, USA; [7]Department of Biostatistics, Harvard School of Public Health, Boston, MA 02215, USA; [9]Current address: Novartis Institutes for Biomedical Research, Center for Proteomic Chemistry, 250 Massachusetts Avenue, Cambridge, MA 02139, USA; [10]Department of Immunology, Genetics and Pathology, Science for Life Laboratory, Rudbeck Laboratory, Uppsala University, 751 85 Uppsala, Sweden

[11]Co-first authors

Supplementary Information

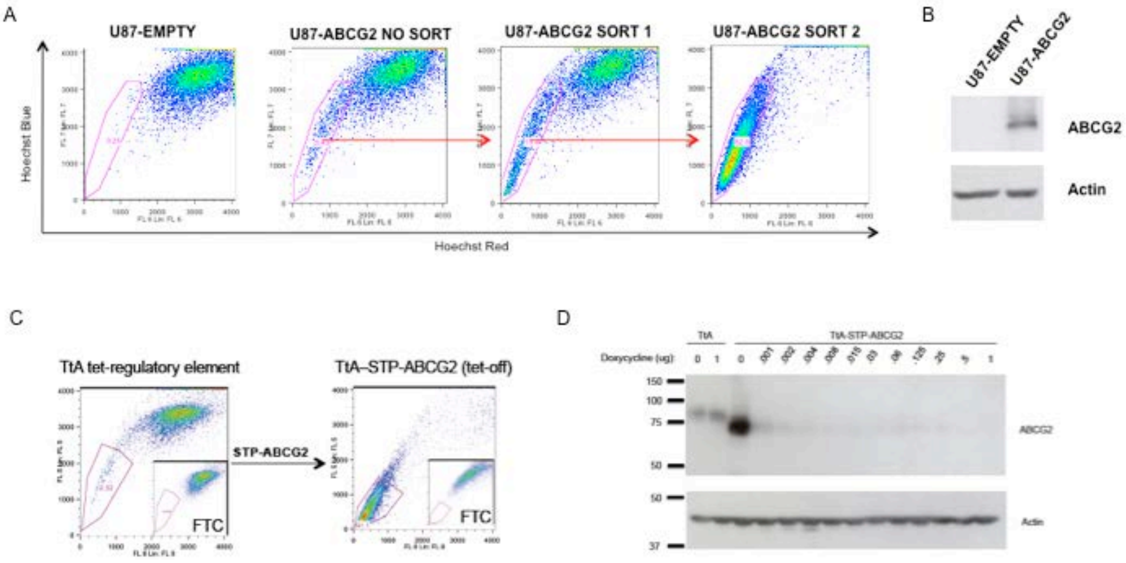

Suppl. Fig. S1. Establishing U87MG cell line with high ABCG2 activity and primary cultures with tet-off-ABCG2. (A-B) U87MG cell line with high ABCG2 activity was generated by retroviral transduction of pbabe-ABCG2 and double sorting for SP fraction. (C-D) Tet-off-ABCG2 was generated by stably expressing tet-regulatory element and tet-off-ABCG2. As low as 1 ng of doxycycline was enough to reduce significantly reduce ABCG2 protein levels.

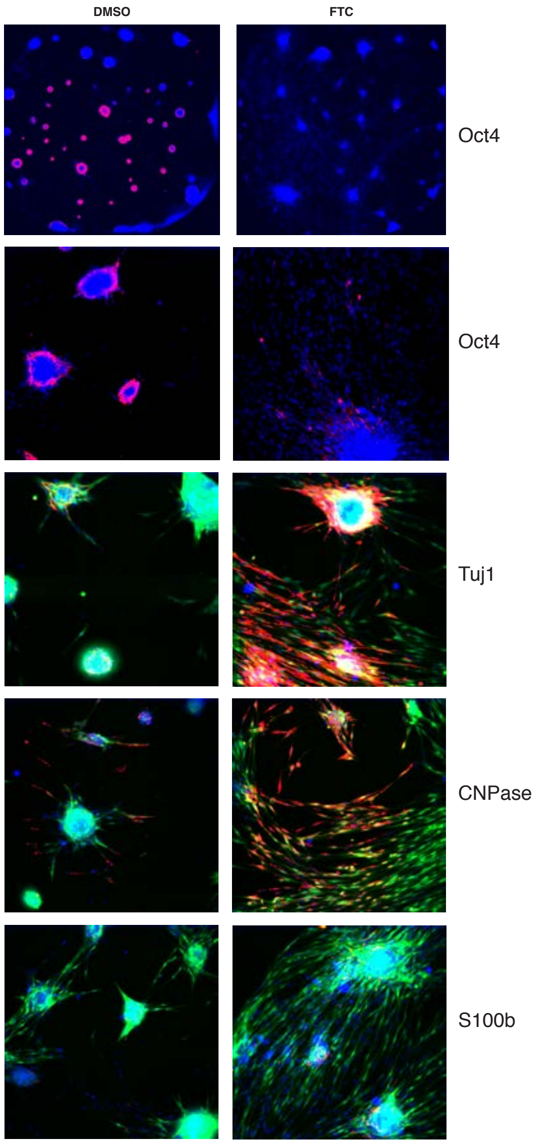

Suppl. Fig. S2. Differentiation and stem cell marker expression following FTC treatment. Additional high-resolution and higher-magnification images of cells presented and studied in Fig. 1g. Blue=DAPI, red=indicated protein, green=GFP (applies to Tuj1, CNPase, S100b).

Suppl. Table S1. List of qRT-PCR primers used in the study

Gene symbol	*Forward Primer 5´->3´*	*Reverse Primer 5´->3´*
MEF/ELF 4 (human)	*5'-TGGAAGGCAGTTTTTTGCTGA-3'*	*5'-GACTTCCGCGGTTGACATG-3'*
SOX2 (human)	*5'-GCCTGGGCGCCGAGTGGA-3'*	*5'-GGGCGAGCCGTTCATGTAGGTCTG-3'*
POU5F1 (human)	*5'-GCTCGAGAAGGATGTGGTCC -3'*	*5'-CGTTGTGCATAGTCGCTGCT -3'*
HES1 (human)	*5'-TGGAAATGACAGTGAAGCACCT-3'*	*5'-GTTCATGCACTCGCTGAAGC-3'*
HEY1 (human)	*5'-CGAAATCCCAAACTCCGATA-3'*	*5'-TGGATCACCTGAAAATGCTG-3'*
HEY2 (human)	*5'-TTGTCAGTATCAGCCACGTC-3'*	*5'-AGTTACCGAGCTGCCTTGAA-3'*
ID1 (human)	*Purchased from Qiagen (QT00230650)*	
ID4 (human)	*5'-GTGCGATATGAACGACTGCT-3'*	*5'-CAGGATCTCCACTTTGCTGA-3'*
18S (human)	*5'-GTAACCCGTTGAACCCCATT-3'*	*5'-CCATCCAATCGGTAGTAGCG-3'*
Mef/Elf4 (mouse)	*5'-TCCTGGATGAGAAGCAGATCTTCA-3'*	*5'-ATGGTGCTGCCTTTGCCATC-3'*

Sox2 (mouse)	*5'-TCCAAAAACTAATCACAACAATCG-3'*	*5'-GAAGTGCAATTGGGATGAAAA-3'*
Pou5f1 (mouse)	*5'-GTTGGAGAAGGTGGAACCAA-3'*	*5'-CTCCTTCTGCAGGGCTTTC-3'*
Hes1 (mouse)	*5'-TGCCAGCTGATATAATGGAGAA-3'*	*5'-CCATGATAGGCTTTGATGACTTT-3'*
Hey1 (mouse)	*5'-CATGAAGAGAGCTCACCCAGA-3'*	*5'-CGCCGAACTCAAGTTTCC-3'*
Id1 (mouse)	*Purchased from Qiagen (QT01743756)*	
Klf4 (mouse)	*5'-GCGGGCTGATGGGCAAGTTTGT-3'*	*5'-TGCCGTCTGGGCTTCCTTTGCT-3'*
cMyc (mouse)	*5'- GACAGCAGCTCGCCCAAAT -3'*	*5'- AGCAGCGAGTCCGAGGAA -3'*
Gapdh (mouse)	*5'-TGGCAAAGTGGAGATTGTTGCC-3'*	*5'-AAGATGGTGATGGGCTTCCCG-3'*
